# Gold-rich ligament nanostructure by dealloying Au-based metallic glass ribbon for surface-enhanced Raman scattering

**DOI:** 10.1038/s41598-017-08033-7

**Published:** 2017-08-08

**Authors:** Bo-Kai Chao, Yi Xu, Hsin-Chia Ho, Pakman Yiu, Yi-Chen Lai, Chan-Hung Shek, Chun-Hway Hsueh

**Affiliations:** 1 Department of Materials Science and Engineering, National Taiwan University, Taipei, 10617 Taiwan; 2 Department of Physics and Materials Science, City University of Hong Kong, Kowloon, Hong Kong

## Abstract

A new method to fabricate an Au-rich interconnected ligament substrate by dealloying the Au-based metallic glass ribbon for surface-enhanced Raman scattering (SERS) applications was investigated in this study. Specifically, three substrates, Au film, Au-based metallic glass ribbon, and dealloyed Au-based metallic glass ribbon, were studied. The dealloyed surface showed ligament nanostructure with protruding micro-islands. Based on the field emission scanning electron microscopy, reflection and scattering measurements, the dealloyed Au-based metallic glass provided a large surface area, multiple reflections, and numerous fine interstices to produce hot spots for SERS enhancements. The SERS signal of analyte, *p*-aminothiophenol, in the micro-island region of dealloyed Au-based metallic glass was about 2 orders of magnitude larger than the flat Au film. Our work provides a new method to fabricate the inexpensive and high SERS enhancements substrates.

## Introduction

Plasmonics has been an important concept in materials science due to the advancement of fabrication technologies in recent several decades. Many applications have been widely used on plasmonics, including solar cells^1^, nonlinear optics^2^, magnetic materials^3^, meta materials^4^, photocatalysts^5^, and surface-enhanced Raman spectroscopy (SERS)^6, 7^. Among those, SERS is a promising technique for molecular detections because the molecular structures can be precisely identified via the considerable amplification of Raman intensity by localized surface plasmon resonance (LSPR), and it has been used in many applications, such as biosensing, chemical imaging^8^, and single molecule detection^9^. For SERS, it has been found that its resonance wavelength strongly depends on the material, size, and shape of nanostructures. For shape effects, many geometrical nanostructures have been discussed, such as disc^10^, ring^11^, and geometrical nanostructures^12, 13^. By using ion lithography or e-beam lithography, accurate nanostructures can be fabricated. However, both ion lithography and e-beam lithography are high-cost and low fabrication efficiency, which become the main limitations of mass production. Therefore, some random nanostructures have been used for SERS substrates, such as nanopillar^14^ and nanoporous substrate^15, 16^. Nevertheless, most of the fabrication processes for nanopillar and nanoporous substrates need to use the high-cost vacuum system and are ineffective for mass production. To avoid using the vacuum system, dealloying homogeneous alloy sheet, including Cu-Zn^17^, Cu-Au^18^, Au-Zn^19^, and Ag-Au^20, 21^ systems, is a low-cost means to fabricate nanoporous substrates. Through removing selected metals from the alloy, the remaining metallic nanoporous structure can be obtained. For SERS applications, by selectively leaching out Ag in the Ag-Au single phase alloy sheet, ultrafine nanoporous Au sheet could be prepared^21^. These fine interstices in the nanoporous structure can serve as hot spots due to near-field coupling of LSPR, and it has demonstrated large SERS enhancements^22^. By applying nanosphere lithography and dealloying on Au-Ag alloy films, Shih *et al*. fabricated nanoporous Au discs, which demonstrated tunable LSPR, 3-dimensional near-field hot-spot distribution, large surface area, and excellent SERS enhancements^23–25^.

Metallic glass is one of the homogeneous metal materials with an amorphous structure. By rapidly quenching the melt to below its glass transition temperature, the frozen atoms promptly solidifies before crystallization. Thus, the amorphous structure can be achieved without crystalline defects and it results in high strength and large elastic strain limit. In addition to the excellent mechanical properties, this amorphous alloy also has the potential for dealloying. The atomic-scale homogeneous disordered structure is expected to produce finer nanopores via dealloying. Au-based metallic glass was the first reported amorphous alloy^26^ which could be a good raw material to fabricate Au nanoporous substrate by removing other elements. In the present study, we developed a new method to fabricate a substrate with a large surface area and Au-rich interconnected ligaments by dealloying the Au-based metallic glass ribbon (Au_55_Cu_25_Si_20_ system) for SERS applications. It is worth noting that dealloying of AuCuAgPdSi metallic glass ribbon has been performed in HNO_3_ solution to result in a nanoporous Au ligament structure, which gave rise to excellent SERS performance using pyridine as probe molecule^27–29^. This strong SERS enhancement was attributed to the localized enhanced electromagnetic fields around nano-sized ligaments, the electromagnetic coupling between ligaments, and the trapped SERS sensitive molecules^27^. Compared with dealloying of crystalline alloys, dealloying of metallic glasses would yield a more uniform nanoporous structure because metallic glasses are free from grain boundaries and other crystalline defects^27^.

## Results

### Dealloying of Au-based metallic glass ribbon

**Figure 1:**
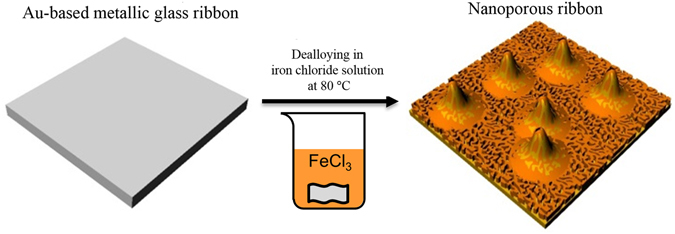
Representation of dealloying method. Au_55_Cu_25_Si_20_ ribbons were prepared by melt-spinning, and the ribbons were used to fabricate Au-rich SERS substrates by chemical dealloying in the iron chloride solution (sigma-aldrich) at 80 °C for 30 min.

**Figure 2:**
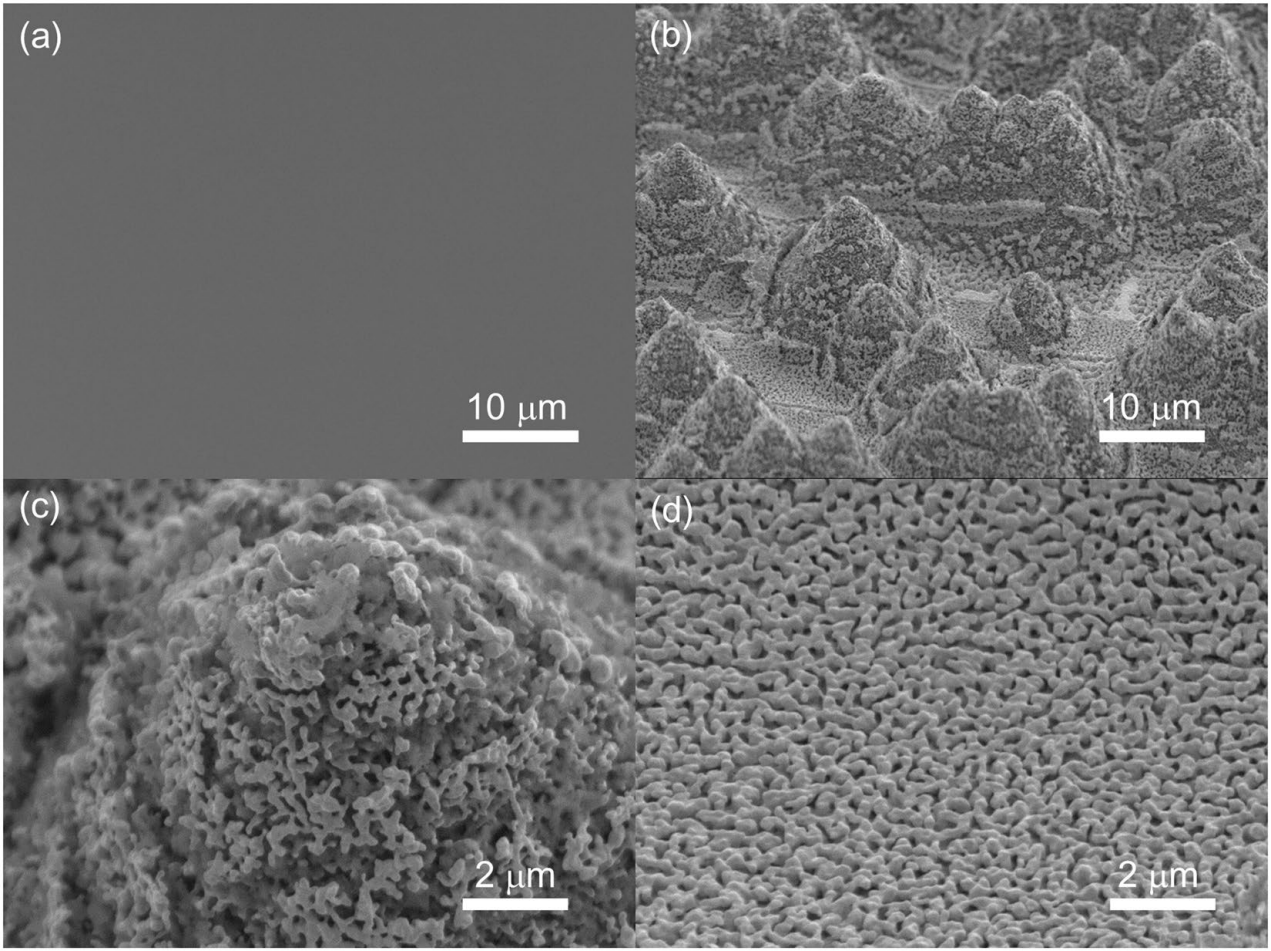
Surface morphologies of Au-based materials. (**a**) Untreated Au-based metallic glass ribbon, (**b**) dealloyed Au-based metallic glass ribbon, and the enlarged (**c**) micro-island region and (**d**) valley region of the dealloyed ligament structure.

The schematic drawing of dealloying of Au-based metallic glass ribbon is shown Fig. 1. The SEM micrographs of the surface morphologies of Au-based materials are shown in Fig. 2. The plane-view of untreated Au-based metallic glass ribbon shown in Fig. 2(a) revealed a flat surface. After dealloying, the Au-based material showed ligament nanostructure with protruding micro-islands in Fig. 2(b). The enlarged micro-island and the flat valley are shown, respectively, in Fig. 2(c) and (d). The diameter of the micro-island was about 10 μm. The widths of ligaments evaluated at their narrower necks in the valley region and the micro-island region were 227 ± 42 and 216 ± 73 nm, respectively, which were obtained by measuring ~80 necks of ligaments. The microstructure and the crystal structure of dealloyed Au-based metallic glass ribbon are shown in Figs S1 and S2 in the Supplementary Information. The micro-island with a larger surface area could adsorb more analyte molecules for SERS enhancements. Compared to dealloying of AuCuAgPdSi metallic glass ribbon^27–29^, our work showed micro-islands to provide a larger surface area in addition to the ligament structure. It is also worth noting that the size of ligament is a function of the Au volume fraction in the as-spun metallic glass ribbon, concentration of dealloying solution, and dealloying temperature and time. However, the parametric study was not performed to optimize the ligament size in the present study.

### Composition and Structure

**Figure 3:**
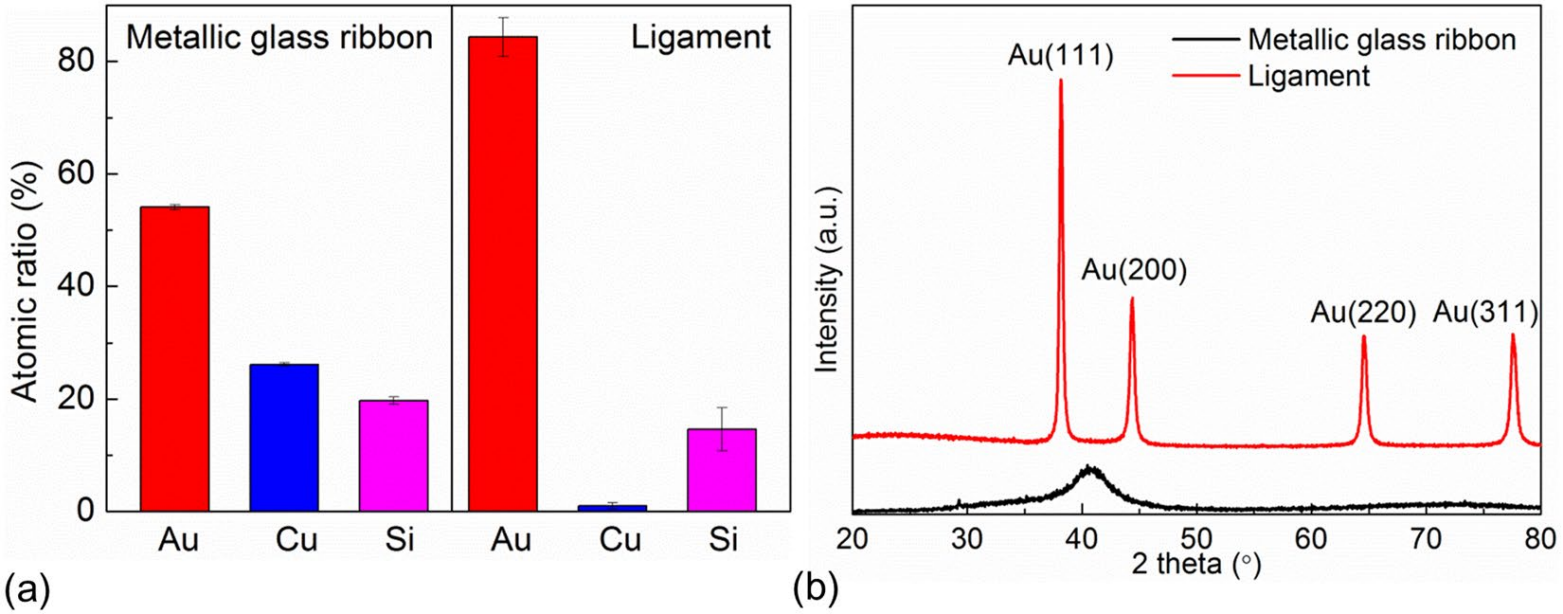
Analysis of the component and structure of Au-based materials. (**a**) The EPMA measurements and (**b**) XRD spectra of Au-based metallic glass ribbon before and after dealloying.

The results of electron probe microanalyzer (EPMA) measurements are shown in Fig. 3(a). The atomic percentages of Au, Cu, and Si were 54.09 ± 0.42%, 26.17 ± 0.32%, and 19.74 ± 0.63%, respectively, for the untreated Au-based metallic glass ribbon, and became 84.37 ± 3.45%, 0.99 ± 0.57%, and 14.64 ± 3.82%, respectively, after dealloying. The changes in the atomic ratio demonstrated that most of Cu and part of Si were removed via dealloying, and the relative Au content increased. The increase of the Au content and the porous structure could result in stronger electromagnetic field enhancements and provide more sites for the adsorption of analyte. It is worth noting that the Si element can be dissolved in alkaline solution, and it might help to further improve SERS detection performance. However, it was not performed in the present study.

The X-ray diffraction (XRD) spectra of Au-based metallic glass ribbon before and after dealloying are shown in Fig. 3(b). The untreated Au-based metallic glass ribbon showed a broad peak for the amorphous structure. The broad peak located at 2*θ* value of about 42° could be the combination of the information of Au_x_Cu_1−x_^30^. The four characteristic peaks of the dealloyed ligament structure located at 2*θ* values of 38.2°, 44.3°, 64.6°, and 77.6° were assigned to Au(111), Au(200), Au(220), and Au(311), respectively, with an ordered face-centered cubic (FCC) structure. This FCC structure of Au was the same as that observed after dealloying of AuCuAgPdSi metallic glass ribbon^27–29^. The XRD data combined with surface morphology observations suggested that crystal nucleated from the amorphous precursor and grew until impingement with neighboring ones to form the ligament structure^28^.

### Optical properties

**Figure 4:**
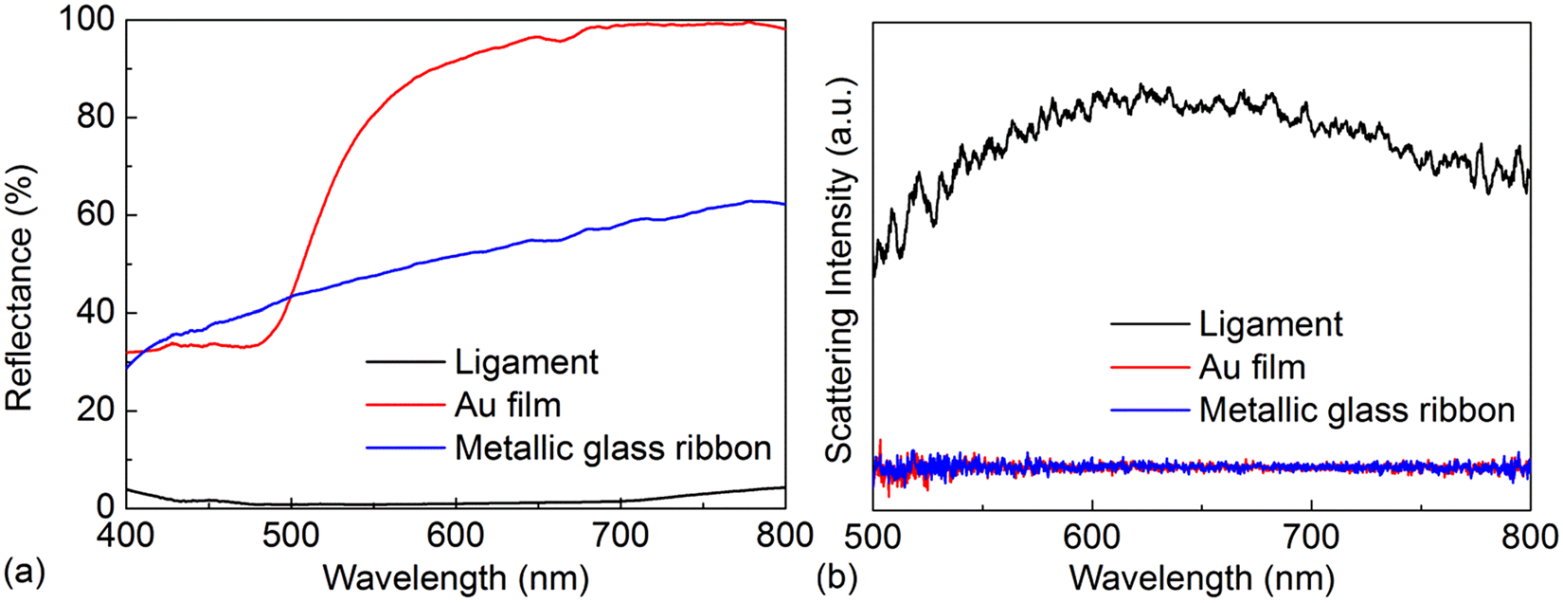
Optical properties of Au-based materials. The measured (**a**) reflectance and (**b**) scattering spectra for Au film, Au-based metallic glass ribbon, and dealloyed Au-based ligament structure.

The measured reflectance spectra of Au film, Au-based metallic glass ribbon, and dealloyed Au-based ligament structure are shown in Fig. 4(a). For Au film, an obvious drop at less than 500 nm was the absorption band due to the interband transitions^31^. For Au-based metallic glass ribbon, the reflectance showed no drop between 400 nm and 800 nm. After dealloying, the dealloyed Au-based ligament structure showed a very low reflectance (less than 4%) in the wavelength range of 400–800 nm. The reason for the extremely low reflectance was the existence of the nanoporous structure, which enabled multiple reflections of incident light to decrease the reflectance. Because the resonance wavelength could not be identified by the reflectance measurements due to the resolution limit of the spectrometer, the scattering spectra were used in addition to the reflectance spectra to identify the resonance wavelength. Using the confocal dark-field microscopy, the scattering spectra are shown in Fig. 4(b). It can be seen that only the scattering spectrum of dealloyed Au-based ligament structure could be detected. The scattering spectra of both Au film and Au-based metallic glass ribbon were very weak because of their planar surfaces. The resonance wavelength of the dealloyed Au-based ligament structure mainly relied on the effective electron oscillation length, which was determined by the pore sizes and gold ligament size^20^. Based on the scattering spectra, the resonance wavelength of dealloyed Au-based ligament structure was located approximately at 622 nm, which served as the basis for selecting the excitation laser wavelength for Raman measurements.

### Raman and SERS measurements

**Figure 5:**
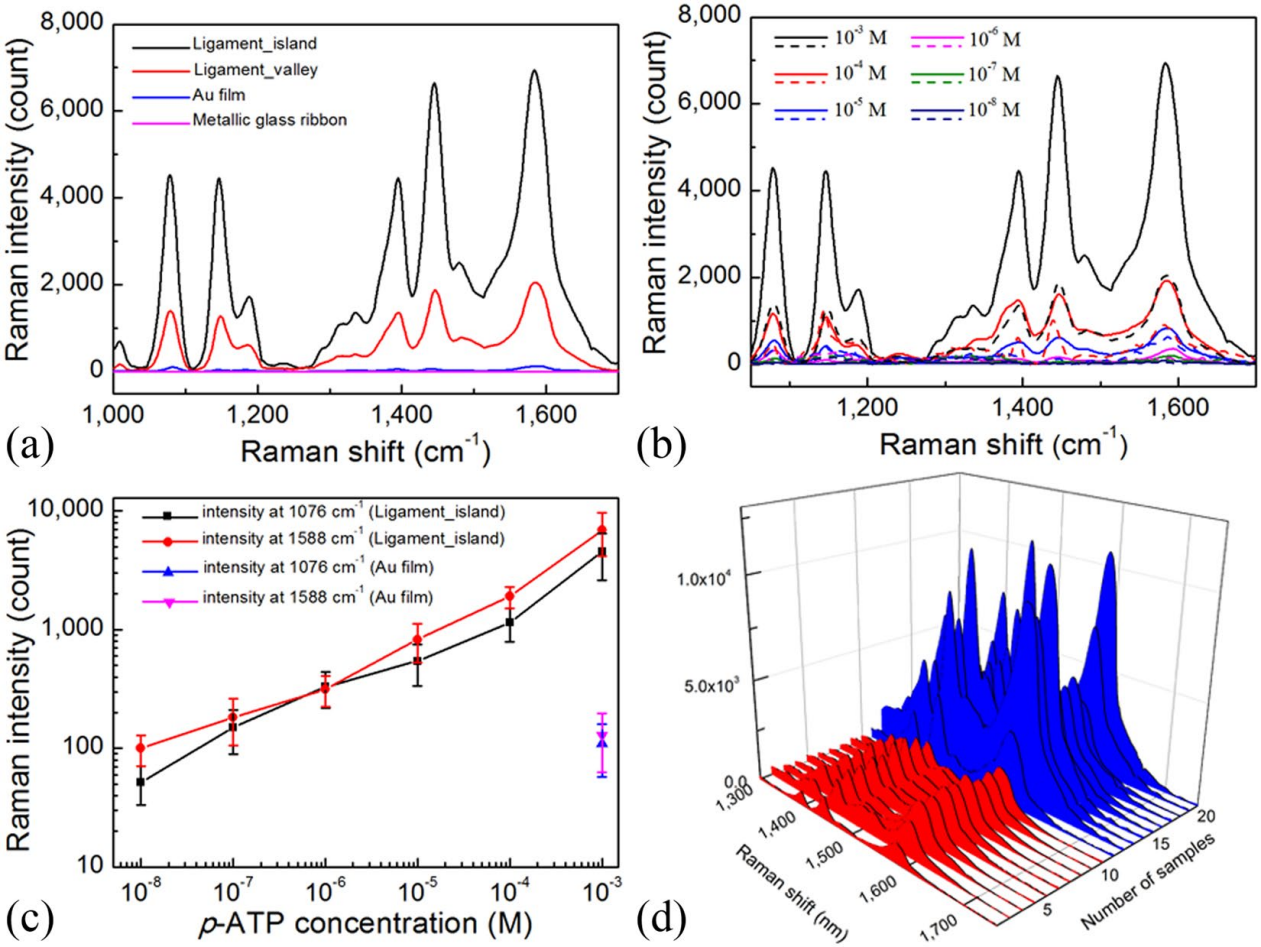
The Raman and SERS measurements. (**a**) Raman spectra of 10^−3^ M *p*-ATP on various substrates, (**b**) SERS spectra of different concentrations of *p*-ATP in the island region (solid line) and the valley region (dashed line) of Au-based ligament structure, (**c**) Raman intensities at 1076 and 1588 cm^−1^ as functions of the *p*-ATP concentration, and (**d**) the reproducibility of SERS spectra in the island and the valley regions of Au-based ligament structure.

The 632.8 nm excitation laser was used for Raman measurements. The average Raman spectra (each spectrum was the average of 10 measured signals) of 10^−3^ M *p*-ATP are shown in Fig. 5(a) for the three different substrates. The results showed that the dealloyed Au-based ligament structure yielded much stronger SERS signals than the Au film because of the existence of hot spots within the fine interstices between the adjacent Au ligament structures. Moreover, the porous structures with low reflectance enabled multiple reflections of the incident laser light as well as provided a larger surface area to contain more *p*-ATP molecules, demonstrating excellent SERS enhancements. Nevertheless, the Raman signal for Au-based metallic glass ribbon was the lowest and could not be detected, which could be due to its low Au content (~54%). Furthermore, the Raman intensity in the island region was stronger than the valley region in the dealloyed Au-based ligament structure because the island region had a larger surface area than the valley region. The corresponding SERS enhancement factor of the dealloyed Au-based ligament structure was calculated in Supplementary Information. This result of the island structure being capable of resulting in more reflections of incident laser light was similar to our previous study of micro-pyramid structure using island lithography^14^.

To find the detection limit of the Au-based ligament structure for SERS applications, the Raman spectra with different concentrations of *p*-ATP on Au-based ligament structure were collected. The Raman spectra (each spectrum was the average of 11 measured signals, see Supplementary Fig. S3) of *p*-ATP on Au-based ligament structure are shown in Fig. 5(b) at different concentrations of *p*-ATP. The solid and the dashed lines correspond to the island and the valley regions, respectively. It could be observed that the Raman intensities decreased with decreasing concentration of *p*-ATP for both regions, while the signals from the island region were always larger than those from the valley region for each *p*-ATP concentration. In Fig. 5(c), the Raman signal still could be detected even at the concentration of *p*-ATP down to 10^−8^ M and the signal was close to the Raman intensity of 10^−3^ M *p*-ATP on the flat Au film.

To examine the reproducibility of SERS spectra of *p*-ATP on dealloyed Au-based ligament structure, several Raman signals were recorded in different regions with a laser spot size of 860 nm diameter. The reproducibility shown in Fig. 5(d) revealed uniform and variable distribution, respectively, in the valley and the island regions, which corresponded to the uniformity of surface morphology. In spite of the variation of measurements from different island regions, the SERS spectra of *p*-ATP in the island region always had a stronger enhancement factor than in the valley region of the dealloyed Au-based ligament structure. The Raman intensity of 10^−3^ M *p*-ATP on the flat Au film is also shown in Fig. 5(c), and the Raman intensity in the micro-island region of dealloyed Au-based metallic glass was about 2 orders of magnitude higher.

## Discussion

In the present study, an Au-rich interconnected ligament substrate with a large surface area was fabricated by dealloying the Au-based metallic glass ribbon. After dealloying, ligament structures with protruding micro-islands were formed on the surface. In the wake of removing Cu and Si, the raise of Au atomic ratio accompanied the crystallization of Au. Also, because of the amorphous structure of the metallic glass, fine nanoporous structure was obtained after dealloying. An excellent detection limit towards 10^−8^ M *p*-ATP mainly resulted from the hot spots produced within the fine interstices between the adjacent Au ligament structures as well as the larger surface area and multiple reflections of incident laser light. Compared with the flat Au substrate, the SERS signal of *p*-ATP in the island region of dealloyed Au-based ligament structure was about 2 orders of magnitude larger than the flat Au film. Also, the micro-island region was more SERS active than the valley region (Fig. 5). Since the size and the composition of ligaments in both regions were similar, it was believed that the morphology governed the SERS enhancements, and the micro-island region had a larger surface area than the valley region.

## Methods

### Sample preparation

The ingots were prepared by alloying the elements Au: 99.99%, Cu: 99.9%, Si: 99.95% purity in an arc-melting furnace. Au_55_Cu_25_Si_20_ ribbons about 50 mm wide and 50 μm thick were prepared by a melt-spinning technique, and a single rotating Cu roller with a surface velocity of 56 m/s was used for melt-spinning. For the as-spun ribbon, the surface on the air-side was shiny and smooth while the surface on the roller-side was dull and rough. The Au_55_Cu_25_Si_20_ ribbons were used to fabricate Au-rich SERS substrate by chemically dealloying in 0.1 M (pH = 0.5) iron chloride solution (sigma-aldrich) at 80 °C for 30 min. Copper was dissolved selectively from the metallic glass ribbon. The as-prepared Au-rich SERS substrates were carefully rinsed with deionized water (18.2 MΩ cm) to remove the residual iron chloride solution. Questions were raised as to whether the difference in surface roughness between the air-side and the roller-side of the as-spun ribbon would result in different microstructures after dealloying and whether the ligament structure after dealloying would be destroyed by polishing before taking SEM micrographs. To address these issues, both the as-spun and the dealloyed ribbons were fractured by bending and the corresponding micrographs are shown in Figs S4 and S5, respectively.

### Analyses

The micrographs of the Au-based materials were obtained using field emission scanning electron microscopy (Nova 450, FEI Co.). The composition of the Au-based materials was analyzed by electron probe microanalyzer (EPMA, JXA-8500F, JEOL) to examine the change of the composition after dealloying. To study the change in structure after dealloying, the X-ray diffractometer (TTRAX 3, Rigaku) was used to examine the crystal structure properties. The phases of Au-based materials were analyzed by examining the diffraction spectrum of the sample via a high energy (18 KW) Cu*K*_*a*_ X-ray (*λ* = 0.15406 nm). The diffraction angle (2*θ*) range was 20° to 80° with a 0.5° incident angle. The structure properties could be used to identify whether crystallization occurred after dealloying. To analyze whether nanoscaled structures existed in the as-spun ribbon, the spectrum of wide-angle X-ray scattering (WAXS) is shown in Fig. S6. The reflectance and the scattering spectra of Au-based materials were collected by the bright-field mode and the dark-field mode, respectively, of Axio Lab A2 optical microscopy (Carl Zeiss Co., Ltd) from a 50× , 0.5-NA objective lens (EC Epiplan-Neofluar) with a halogen white light source (Carl Zeiss Co., Ltd) to examine the surface plasmon resonance wavelength of Au-based materials. The measured wavelength range of reflectance and scattering spectra were between 400 and 700 nm with a spot size of about 10 μm.

### Raman and SERS measurements

A self-assembled monolayer of *p*-aminothiophenol (*p*-ATP, Merck, 99.0%) was used as the probing molecule to obtain the Raman and SERS spectra. The Au-based materials were soaked in the freshly prepared *p*-ATP solution with a concentration of 10^−3^ M at room temperature for 24 hours. The *p*-ATP molecules were self-assembly bounded with Au-based material via the thiol−gold conjugation chemistry. To avoid the nonuniform distribution from physical adsorption of residual molecules, the sample were rinsed in a solution containing 90% deionized water and 10% ethanol to remove the residual molecules. Finally, the sample was dried with flow N_2_. The Raman and SERS spectra were obtained using a Micro-Raman spectrometer (iHR550, HORIBA). The wavelength of excitation laser and the laser beam diameter were 632.8 nm and 860 nm, respectively. Through a 50× objective lens, the laser power reached the sample surface was about 1 mW.

## Electronic supplementary material

Supplementary Information
