# Supplementary material for: Gold-rich ligament nanostructure by dealloying Au-based metallic glass ribbon for surface-enhanced Raman scattering
Source: Sci Rep. 2017 Aug 8;7:7485. doi: 10.1038/s41598-017-08033-7 (PMC5548893; doi:10.1038/s41598-017-08033-7)
Supplement: Supplementary file 1 — Supplementary Information [file 41598_2017_8033_MOESM1_ESM.pdf]

Supplementary Information

Gold-rich ligament nanostructure by dealloying Au-based metallic glass ribbon for surface-enhanced Raman scattering

Bo-Kai Chao[1], Yi Xu[2], Hsin-Chia Ho[1], Pakman Yiu[2], Yi-Chen Lai[1], Chan-Hung Shek[2] and Chun-Hway Hsueh[1]*

[1]Department of Materials Science and Engineering, National Taiwan University, Taipei 10617, Taiwan

[2]Department of Physics and Materials Science, City University of Hong Kong, Kowloon, Hong Kong

*e-mail: hsuehc@ntu.edu.tw

Microstructure and crystal structure of dealloyed Au-based metallic glass ribbon

After chemical dealloying of the Au-based metallic glass ribbon in the iron chloride solution, the sample was cold mounted, polished, and then examined by SEM. The SEM micrograph of the cross-section of dealloyed ribbon is shown in Fig. S1(a). Micro-islands with height about 10 μm protruding from the surface of ribbon could be observed. It is also noted that the top region (encircled by the red square) of micro-island exhibited a polycrystalline structure, while the bottom region (encircled by the yellow square) close to the un-dealloyed region showed a single-crystal feature, which were confirmed by the SAED patterns shown in Fig. S1(b) and (c), respectively. The crystal structure of the un-dealloyed region could not be determined directly by SAED in TEM because the thickness exceeded the resolution limit. However, it could be inferred from the XRD pattern by comparing the crystal structure of as-spun ribbon with the ribbon dealloyed for 5 min, as shown in Fig. S2. It is apparent that after the short duration of dealloying, the broad peak for the amorphous structure of ribbon located at 2θ value of about 42° coexisted with the characteristic peaks of gold, and it implied that the un-dealloyed region underneath the crystalline micro-islands retained the amorphous phase.

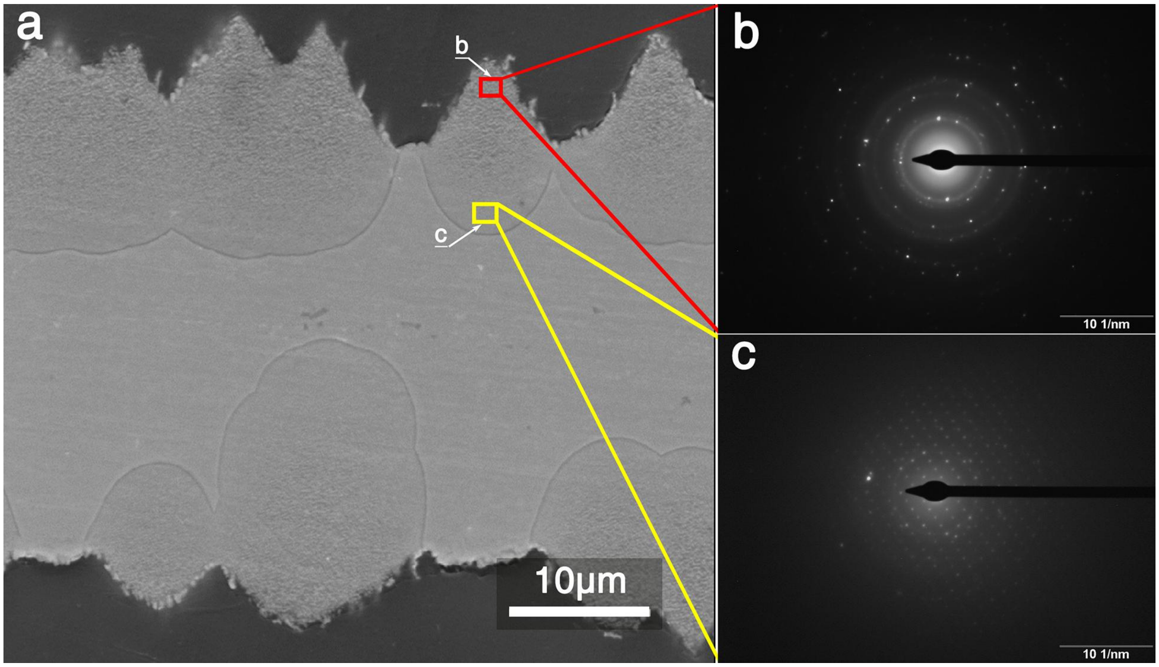

Figure S1. (a) SEM micrograph of the cross-section of dealloyed Au-based metallic glass ribbon and SAED patterns corresponding to the regions encircled by (b) the red square and (c) the yellow square.

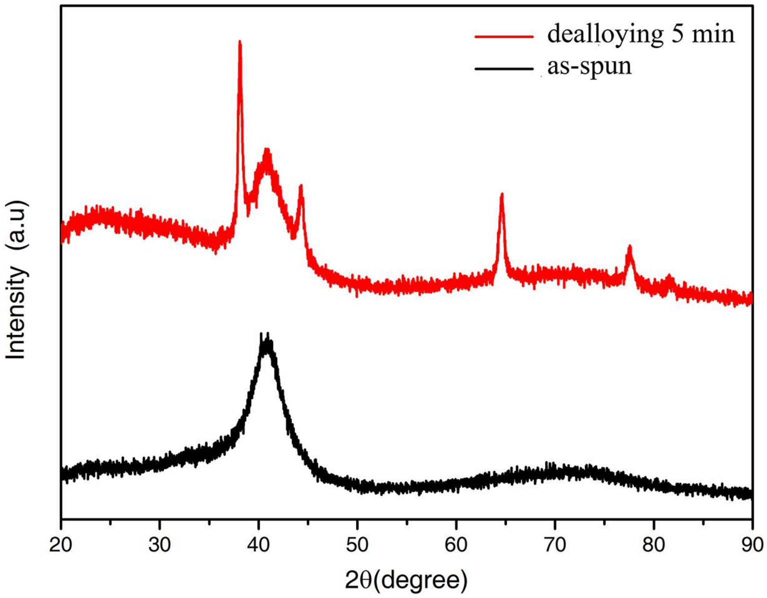

Figure S2. XRD patterns of the as-spun Au-based metallic glass ribbon and ribbon dealloyed for 5 min.

SERS enhancement factor

The SERS enhancement factor, *EF*, was obtained from[1]

$$EF = \frac{I_{SERS}}{N_{SERS}} \frac{N_{BULK}}{I_{BULK}} \quad \text{(S1)}$$

where I_{SERS} and I_{BULK} are the intensities of the SERS and normal Raman spectra, respectively, of *p*-ATP at the same Raman band of 1076 cm^{-1}, and N_{SERS} and N_{BULK} are the numbers of *p*-ATP molecules illuminated under the laser focus spot in SERS and bulk measurements, respectively. The N_{BULK} value of 6.59×10^9 could be calculated from $Ah\rho/M$, where A is the spot size of laser (0.86 μm), h is the penetration depth (2 μm), ρ is the density (1.18 g/cm^3), and M is the molecular weight of *p*-ATP (125.19 g/mol)[2]. N_{SERS} could be obtained from

$$N_{SERS} = S_r \times N_{PLANAR} \quad \text{(S2)}$$

where S_r is the surface area ratio between the SERS substrate and the planar substrate obtained from AFM measurements. The scanned area in AFM for the Au film (S_r=1.00) and the as-spun metallic glass ribbon (S_r=1.01) were chosen at arbitrary position due to the uniform morphology, while the micro-island region was scanned for the dealloyed ligament structure (S_r=2.21). N_{PLANAR} is the number of *p*-ATP molecules on the planar Au film assuming that the Au film is uniformly covered by monolayer self-assembly *p*-ATP molecules. The SERS enhancement factors of as-spun metallic glass ribbon, Au film, and micro-island region of the dealloyed ligament structure calculated from equations (S1) and (S2) were 3.51, 1.32×10^1, and 9.86×10^4, respectively.

Raman spectra of *p*-ATP with different concentrations

To find the detection limit of the Au-based ligament structure for SERS applications, the Raman spectra with different concentrations of *p*-ATP on Au-based ligament structure were collected. The Au-based ligament structures were soaked in the freshly prepared *p*-ATP solution with different concentrations at room temperature for 24 h. The 632.8 nm excitation laser was used for Raman measurements. Fig. S3 shows the Raman spectra of *p*-ATP with different concentrations of 10^{-3} M, 10^{-4} M, 10^{-5} M, 10^{-6} M, 10^{-7} M, and 10^{-8} M collected from the micro-island region. The measurements were performed at 11 different locations for each concentration.

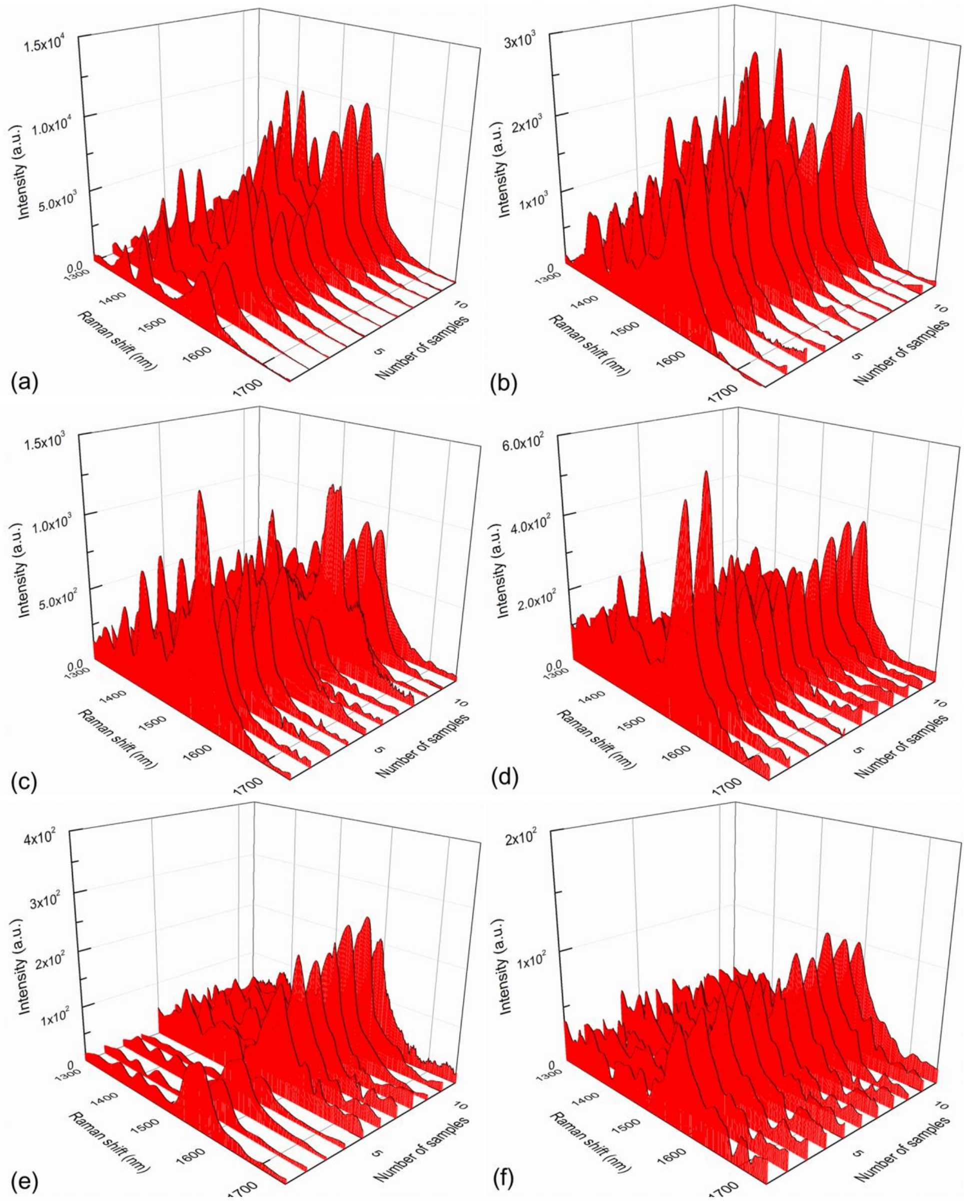

Figure S3. The Raman spectra of *p*-ATP with different concentrations collected from the micro-island region: (a) 10^{-3} M, (b) 10^{-4} M, (c) 10^{-5} M, (d) 10^{-6} M, (e) 10^{-7} M, and (f) 10^{-8} M.

Microstructure of ribbon without polishing

Because the ligament structure and related information could possibly be destroyed by the cold mount and polishing processes, the ribbon was fractured by bending without other treatments to reveal the microstructures of the pristine cross-section and air-side and roller-side surfaces of the ribbon. The tilted-view SEM micrographs of as-spun metallic glass ribbon of the cross-section with the air-side surface and the roller-side surface are shown in Fig. S4(a) and (b), respectively. While the air-side surface showed relatively smooth morphology, the roller-side

surface was relatively rough due to the contact with Cu roller during the melt-spinning process. After dealloying, the tilted-view SEM micrograph of the dealloyed ribbon is shown in Fig. S5, and similar protruding micro-islands were observed on both sides of the dealloyed ribbon, indicating the independence of the protrusion mechanism on the surface roughness of the ribbon.

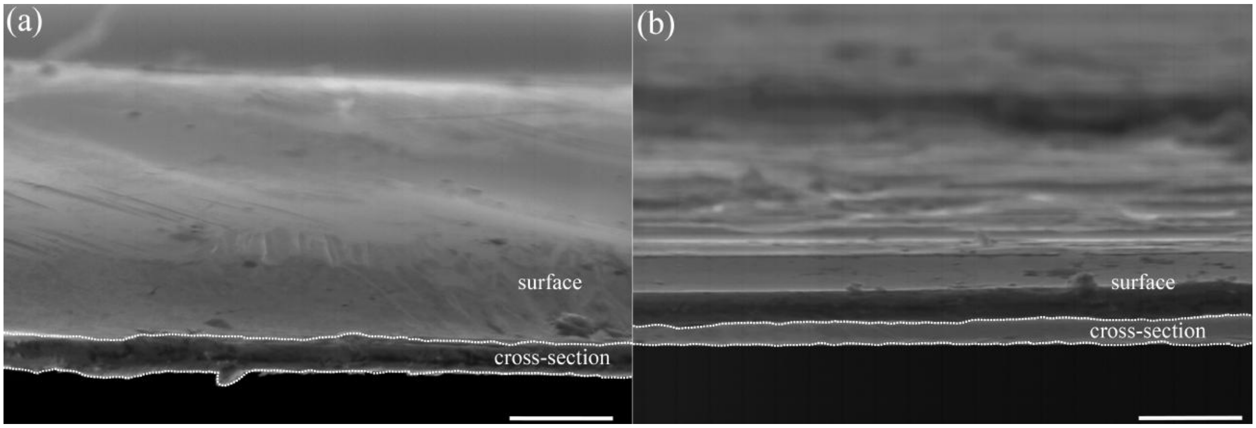

Figure S4. Tilted-view SEM micrograph of as-spun ribbon showing (a) cross-section and air-side surface and (b) cross-section and roller-side surface. The scale bar is 10 μm.

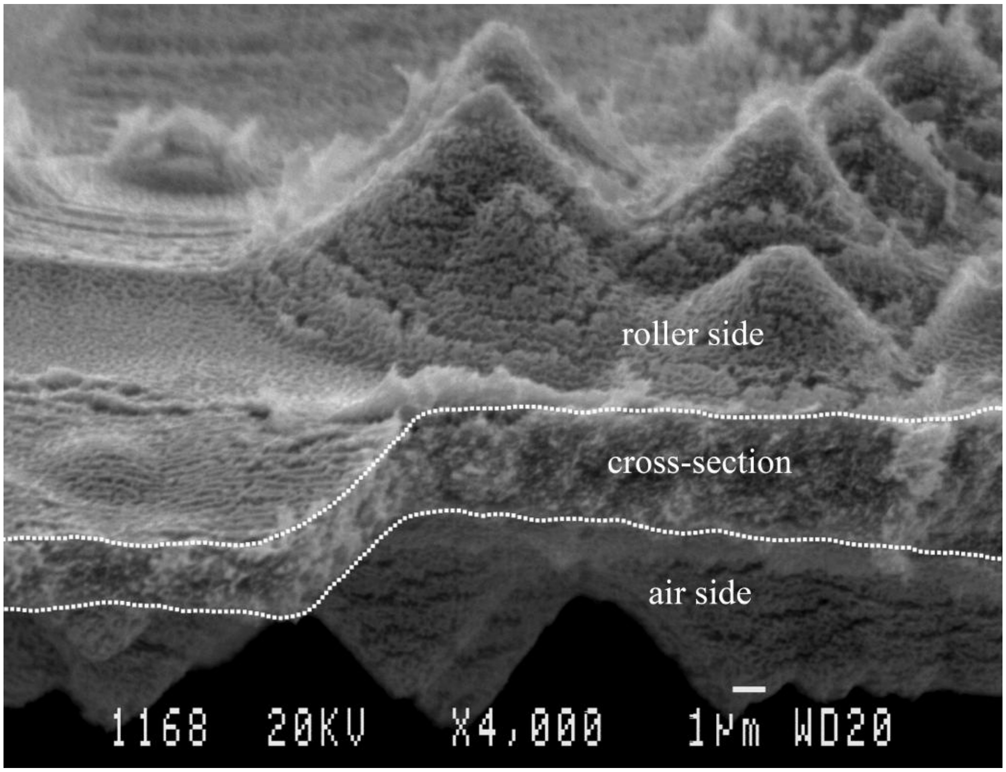

Figure S5. Tilted-view SEM micrograph of dealloyed metallic glass ribbon.

Wide-angle X-ray scattering

In order to analyze whether nanoscaled structures existed in the as-spun ribbon, wide-angle X-ray scattering (WAXS, SAXSpace, Anton Paar, Austria) was performed and the spectrum is shown in Fig. S6. Only amorphous signal and no crystalline phase could be identified. Hence, protrusion of micro-islands after dealloying could not result from removal of the crystalline phase, which could exist in a defective amorphous ribbon. Also, if there were nanocrystals in the as-spun metallic glass ribbon, the dealloying process would proceed layer by layer instead of the formation of protruding micro-islands. Since the bottom of cone-shaped protrusion shown in Fig. S1(a) was determined to be a single crystal. The dissolution might penetrate the ribbon surface at specific sites, in which the metallic glass turned into crystallized grains. Subsequently, nanoscaled pores and ligaments grew normal to the curved grain boundary to form the protruding micro-island.

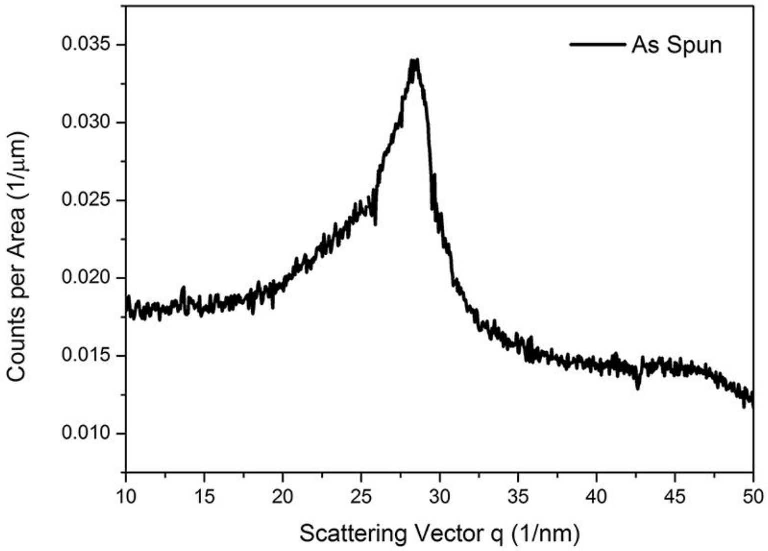

Figure S6. Wide-angle X-ray scattering spectrum of the as-spun ribbon.

References

1. Chao, B. K., Cheng, H. H., Nien, L. W., Chen, M. J., Nagao, T., Li, J. H. & Hsueh, C. H. Anti-reflection textured structures by wet etching and island lithography for surface-enhanced raman spectroscopy. *Appl. Surf. Sci.* **357,** 615-621 (2015).
2. Liu, X., Cao, L., Song, W., Ai, K. & Lu, L. Functionalizing metal nanostructured film with graphene oxide for ultrasensitive detection of aromatic molecules by surface-enhanced Raman spectroscopy. *ACS Appl. Mater. Interfaces* **3,** 2944-2952 (2011).
